# Biological Effects of PMMA and Composite Resins on Human Gingival Fibroblasts: An In Vitro Comparative Study

**DOI:** 10.3390/ijms25094880

**Published:** 2024-04-30

**Authors:** Ylenia Della Rocca, Enrico Matteo Traini, Oriana Trubiani, Tonino Traini, Antonella Mazzone, Guya Diletta Marconi, Jacopo Pizzicannella, Francesca Diomede

**Affiliations:** 1 Department of Innovative Technologies in Medicine & Dentistry, University “G. d’Annunzio” Chieti-Pescara, Via dei Vestini, 31, 66100 Chieti, Italy; ylenia.dellarocca@unich.it (Y.D.R.); trainienrico@gmail.com (E.M.T.); oriana.trubiani@unich.it (O.T.); tonino.traini@unich.it (T.T.); antonella.mazzone@unich.it (A.M.); francesca.diomede@unich.it (F.D.); 2 Department of Engineering and Geology, University “G. d’Annunzio” Chieti-Pescara, Viale Pindaro, 42, 65127 Pescara, Italy; jacopo.pizzicannella@unich.it

**Keywords:** biocompatibility, inflammosome, prosthetic resins, human gingival fibroblasts

## Abstract

The use of temporary resin for provisional restorations is a fundamental step to maintain the position of prepared teeth, to protect the pulpal vitality and the periodontal health as well as the occlusion. The present study aimed at evaluating the biological effects of two resins used in dentistry for temporary restorations, Coldpac (Yates Motloid) and ProTemp 4™ (3M ESPE ™), and their eluates, in an in vitro model of human gingival fibroblasts (hGFs). The activation of the inflammatory pathway NFκB p65/NLRP3/IL-1β induced by the self-curing resin disks was evaluated by real-time PCR, Western blotting and immunofluorescence analysis. The hGFs adhesion on resin disks was investigated by means of inverted light microscopy and scanning electron microscopy (SEM). Our results suggest that hGF cells cultured in adhesion and with eluate derived from ProTemp 4™ (3M ESPE ™) resin evidenced a downregulation in the expression of the inflammatory mediators such as NFκB p65, NLRP3 and IL-1β compared to the cells cultured with Coldpac (Yates Motloid) after 24 h and 1 week of culture. Furthermore, the cells cultured with ProTemp 4™ (3M ESPE ™) after 24 h and 1 week of culture reported a higher cell viability compared to the cells cultured with Coldpac (Yates Motloid), established by MTS cell analysis. Similar results were obtained when hGFs were placed in culture with the eluate derived from ProTemp 4™ (3M ESPE ™) resin which showed a higher cell viability compared to the cells cultured with eluate derived from Coldpac (Yates Motloid). These results highlighted the lower pro-inflammatory action and improved cell biocompatibility of ProTemp 4™ (3M ESPE ™), suggesting a better performance in terms of cells–material interaction.

## 1. Introduction

Over the years dentistry has seen a consolidation of the resinous materials use [1]. In prosthodontics there are multiple uses, such as denture bases, denture frameworks and temporary restorations. In the conservative field, these materials can be used both in the restoration of elements affected by caries, such as in the case of composite, and in the aesthetic field, such as pre-visualization of the result through the so called “mockup”. Among the various uses of composite resins, provisional restorations stand out as a fundamental step in prosthetic treatment plans. In the design and realization of the provisional prosthesis, different types of resin materials can be used, each with peculiar characteristics depending on the structure and molecular composition [2]. Polymethyl methacrylate (PMMA) has been one of the most used acrylic polymers in dentistry for almost a century. Acrylic polymers are easily moldable, colorable, and capable of reproducing both teeth and oral mucosa [3]. In the PMMA formulation, different materials such as carbon fibers, glass fibers and polyethylene can be added to increase the mechanical resistance [4]. In the biomedical field of bone regeneration, PMMA is used in the bone cement composition as a vicle for drugs and antibiotics or as a support for hydroxyapatite [5,6]. PMMA can be composed of polymer powder and monomer liquid, which can be polymerized at cold, hot or with photo-polymerizing light [7]. In addition, there are formulations with pre-polymerized PMMA billets that are polymerized under high temperatures and pressure values, to be used with CAD/CAM procedures [8]. However, PMMA has the disadvantage of having a low fatigue resistance and releasing residual monomer, which can lead to irritation or allergy to the oral mucosa [9]. Another well-known composite resin base used in dentistry is bisphenol A glycidyl methacrylate (Bis-GMA). Thanks to its characteristics and the possibility of being used together with other monomers to improve its properties, Bis-GMA can be found as the base of different resins employed in different fields such as orthodontics, endodontics, prosthodontics and restorative dentistry. In orthodontics, Bis-GMA-based resins are mainly used to connect the orthodontic brackets to the buccal surface of the teeth after etching and adhesive application. In endodontics, it can be the basis of the root canal cements used in the endodontic seal. In prosthetic and restorative fields, Bis-GMA composites can be used for temporary crowns and bridges, construction of mockups, and reconstruction of teeth abutments. Moreover, it is used for veneers and teeth fillings. Layered over metallic frameworks, it can be used for long-lasting temporary restorations [10,11,12]. Nevertheless, the main problem with these materials is related to the incomplete polymerization process and the leakage of monomers following the corrosion process under mechanical strain, oral secretions and pH change [13,14]. In addition, it is reported in the literature that adverse reactions can be caused by the improper use of dental resins [15]. Previous reports showed that dental resins could alter the cell viability, metabolic activity, and cell morphology of human gingival fibroblasts (hGFs) [16,17]. Furthermore, other studies evaluated the effects of the resin eluates in preclinical models [18,19,20,21,22]. These studies highlight how cells may react to a solubilization of different types of monomers within the oral cavity and their biological effects.

Moreover, oral resin may trigger inflammation. The NFκB p65/NALP3/IL-1β inflammatory pathway is a signaling pathway that involves the transcription factor nuclear factor kappa-light-chain-enhancer of activated B cells (NFκB), the inflammasome protein NOD-, LRR- and pyrin domain-containing 3 (NLRP3), and the pro-inflammatory cytokine interleukin-1 beta (IL-1β). The NFκB p65/NALP3/IL-1β pathway regulates various aspects of innate and adaptive immune functions and inflammatory responses [23,24]. In addition, Ki-67 was also analyzed in order to understand the cell proliferation effect. Ki-67 is a proliferation marker that was identified in Hodgkin lymphoma cell nuclei [25]. Based on the literature, this protein plays a role in cell cycle regulation, heterochromatin maintenance, and assembly of the peri chromosomal layer on mitotic chromosomes [26,27,28]. The present study was aimed at investigating the biocompatibility and the cell adhesion capability of the provisional resins and their eluates using an in vitro model of hGFs. The biological effects of disks and their eluates made with Bis-GMA-based resin (ProTemp 4™ (3M ESPE ™)) and PMMA-based resin (Coldpac Yates Motloid) have been evaluated.

## 2. Results

### 2.1. Cell Viability and Proliferation Evaluation

**Figure 1:**
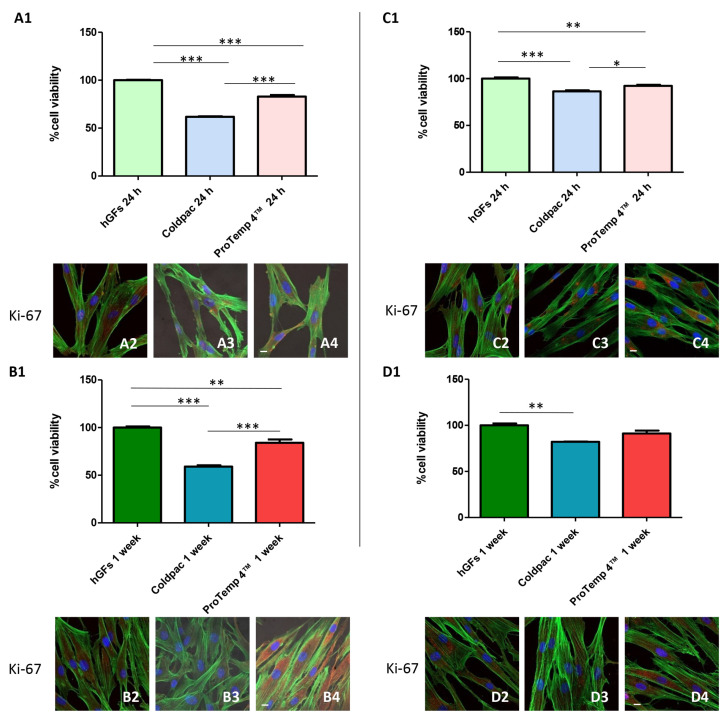
(**A1**,**B1**) The cell metabolic activity of hGFs cultured alone, hGFs cultured with Coldpac (Yates Motloid) and hGFs cultured with ProTemp 4™ (3M ESPE ™) for 24 h and 1 week. (**C1**,**D1**) The cell metabolic activity of hGFs alone, hGFs cultured with eluate derived from Coldpac (Yates Motloid) and hGFs cultured with eluate derived from ProTemp 4™ (3M ESPE ™) for 24 h and 1 week. Ki-67 immunofluorescence in hGFs cultured alone (**A2**,**B2**), hGFs cultured with Coldpac (Yates Motloid) (**A3**,**B3**) and hGFs cultured with ProTemp 4™ (3M ESPE ™) (**A4**,**B4**) for 24 h and 1 week. (Confocal microscopy. Red fluorescence: Ki-67; green fluorescence: actin; blue fluorescence: nuclei). Ki-67 immunofluorescence in hGFs cultured alone (**C2**,**D2**), hGFs cultured with eluate derived from Coldpac (Yates Motloid) (**C3**,**D3**) and hGFs cultured with eluate derived from ProTemp 4™ (3M ESPE ™) (**C4**,**D4**) for 24 h and 1 week. (Confocal microscopy. red fluorescence: Ki-67; green fluorescence: actin; blue fluorescence: nuclei). Scale bar: 20 μm. * *p* < 0.05; ** *p* < 0.01; *** *p* < 0.001.

Cell viability was analyzed by MTS assay on hGFs alone, hGFs cultured with Coldpac (Yates Motloid), and hGFs cultured with ProTemp 4™ (3M ESPE ™) and their eluates for 24 h and 1 week (Figure 1). The results showed that cell viability was significantly decreased in hGFs cultured with Coldpac (Yates Motloid) when compared to hGFs cultured alone or with ProTemp 4™ (3M ESPE ™) at both time points (Figure 1(A1,B1)). In parallel, the cell viability was also evaluated in hGFs alone, hGFs cultured with the eluate derived from Coldpac (Yates Motloid) and hGFs cultured with eluate derived from Protemp 4™ (3M ESPE ™). The results obtained are comparable with the results mentioned above in terms of cell viability (Figure 1(C1,D1)). Furthermore, Ki-67, a cellular marker for proliferation evaluated by immunofluorence reported a higher expression in hGFs cultured with ProTemp 4™ (3M ESPE ™) and its eluate compared to the Coldpac (Yates Motloid) culture and its eluate (Figure 1A1–D1).

### 2.2. hGFs Alone, Cultured with Coldpac (Yates Motloid) and Cultured with ProTemp 4™ (3M ESPE ™) Morphological Analysis

**Figure 2:**
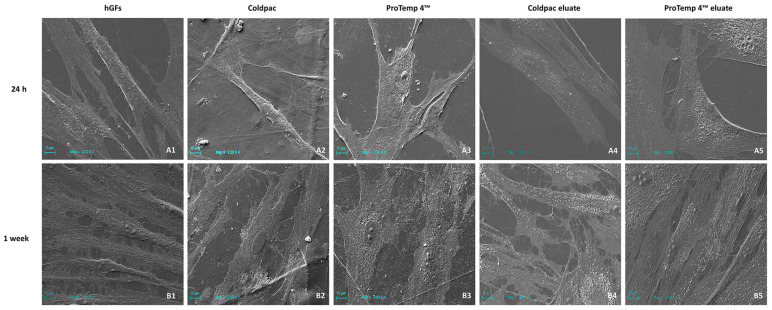
Representative SEM images of: (**A1**) hGFs cultured alone, (**A2**) hGFs cultured with Coldpac (Yates Motloid) resin, (**A3**) hGFs cultured with ProTemp 4™ (3M ESPE ™) resin, (**A4**) hGFs cultured with Coldpac (Yates Motloid) eluate, and (**A5**) hGFs cultured with ProTemp 4™ (3M ESPE ™) eluate for 24h. (**B1**) hGFs cultured alone, (**B2**) hGFs cultured with Coldpac (Yates Motloid) resin, (**B3**) hGFs cultured with ProTemp 4™ (3M ESPE ™) resin, (**B4**) hGFs cultured with Coldpac (Yates Motloid) eluate, and (**B5**) hGFs cultured with ProTemp 4™ (3M ESPE ™) eluate for 1 week. Scale bar 10 μm.

After 24 h and 1 week, the morphology of hGFs cultured alone, with Coldpac (Yates Motloid) and with ProTemp 4™ (3M ESPE ™) were studied using SEM (Figure 2). HGFs adherent on a plastic surface showed a fibroblast-like morphology, and after 1 week of culture exhibited numerous cytoplasmic processes in contact with neighbouring cells; nuclei and nucleoli were also evident. After one week of culture, cells cultured on Coldpac (Yates Motloid) showed different morphological features, while the hGFs cultured on ProTemp 4™ (3M ESPE ™) displayed a similar morphology as control cells evidencing nuclei and nucleoli. No significant morphological differences were found by SEM analysis of the hGFs cultured with the Coldpac (Yates Motloid) and Protemp (3M ESPE ™) eluate at 24 h and 1 week compared to the control.

### 2.3. CLSM and Western Blot Analyses

**Figure 3:**
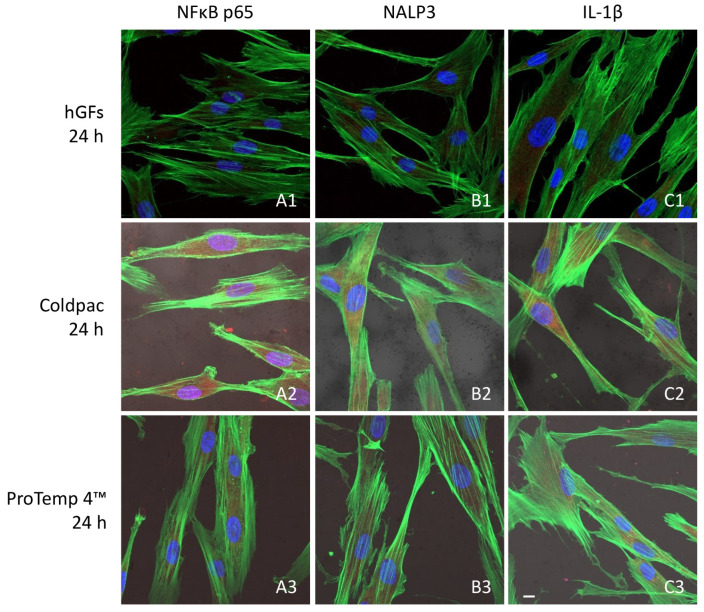
NFκB p65/NLRP3/IL-1β expression analyzed by confocal microscopy. Expression of NFκB p65 (**A1**–**A3**), NLRP3 (**B1**–**B3**) IL-1β (**C1**–**C3**) were evaluated in hGFs cultured alone, hGFs cultured with Coldpac (Yates Motloid), and hGFs cultured with ProTemp 4™ (3M ESPE ™) for 24 h. Red fluorescence: NFκB p65/NLRP3/IL-1β; green fluorescence: actin; blue fluorescence: nuclei. Scale bar: 20 μm.

**Figure 4:**
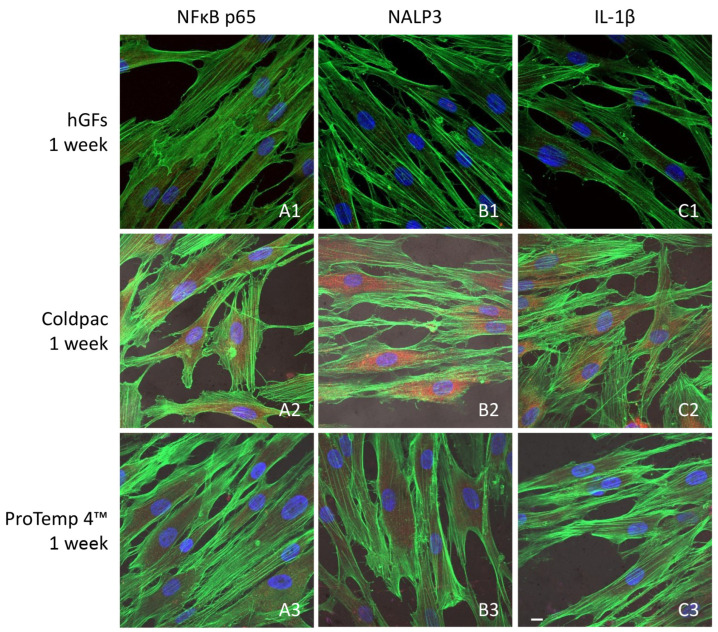
NFκB p65/NLRP3/IL-1β expression analyzed by confocal microscopy. Expression of NFκB p65 (**A1**–**A3**), NLRP3 (**B1**–**B3**) IL-1β (**C1**–**C3**) were evaluated in hGFs cultured alone, hGFs cultured with Coldpac (Yates Motloid) and hGFs cultured with ProTemp 4™ (3M ESPE ™) for 1 week. Red fluorescence: NFκB p65/NLRP3/IL-1β; green fluorescence: actin; blue fluorescence: nuclei. Scale bar: 20 μm.

**Figure 5:**
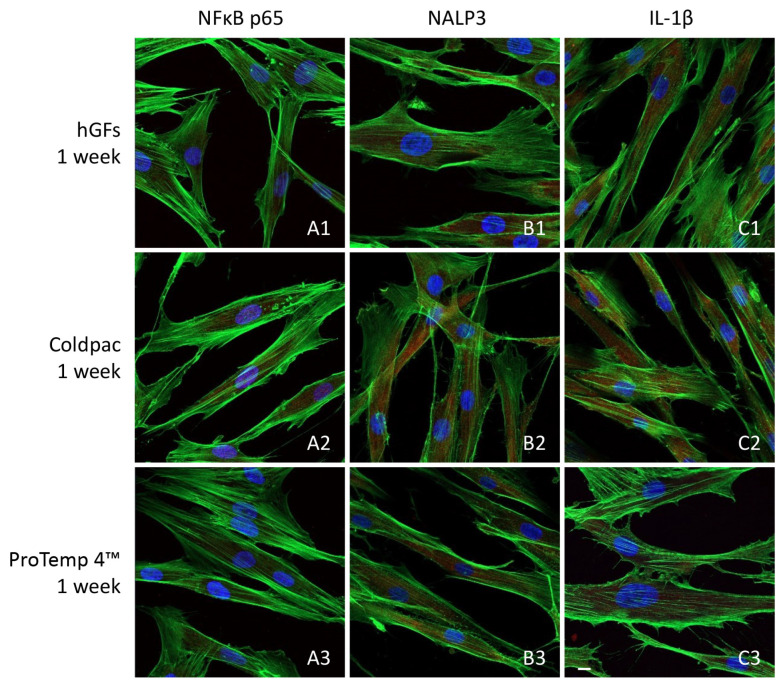
NFκB p65/NLRP3/IL-1β expression analyzed by confocal microscopy. Expression of NFκB p65 (**A1**–**A3**), NLRP3 (**B1**–**B3**) IL-1β (**C1**–**C3**) were evaluated in hGFs cultured alone, hGFs cultured with eluate derived by Coldpac (Yates Motloid) and hGFs cultured with eluate derived from ProTemp 4™ (3M ESPE ™) for 24 h. Red fluorescence: NFκB p65/NLRP3/IL-1β; green fluorescence: actin; blue fluorescence: nuclei. Scale bar: 20 μm.

**Figure 6:**
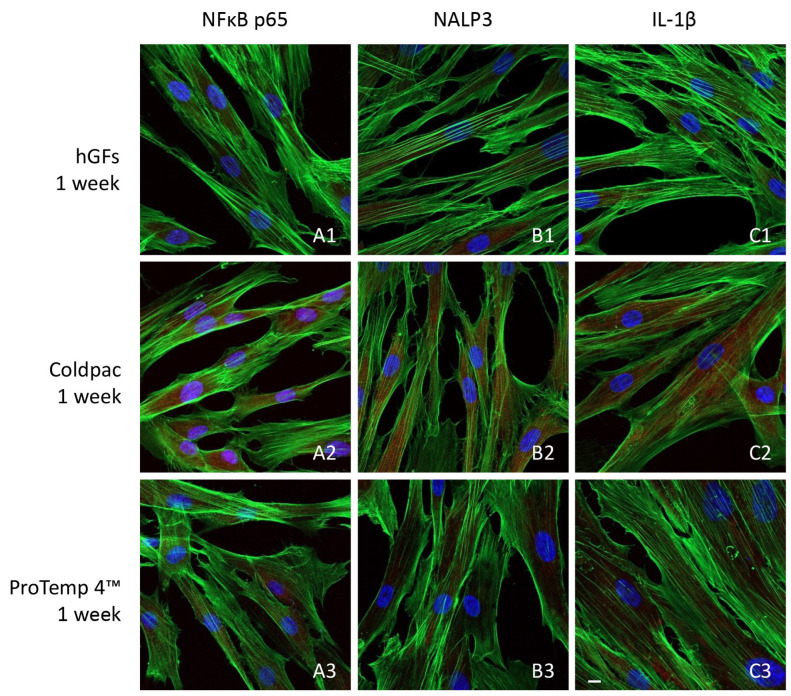
NFκB p65/NLRP3/IL-1β expression analyzed by confocal microscopy. Expression of NFκB p65 (**A1**–**A3**), NLRP3 (**B1**–**B3**) IL-1β (**C1**–**C3**) were evaluated in hGFs cultured alone, hGFs cultured with eluate derived from Coldpac (Yates Motloid) and hGFs cultured with eluate derived from ProTemp 4™ (3M ESPE ™) for 1 week. Red fluorescence: NFκB p65/NLRP3/IL-1β; green fluorescence: actin; blue fluorescence: nuclei. Scale bar: 20 μm.

**Figure 7:**
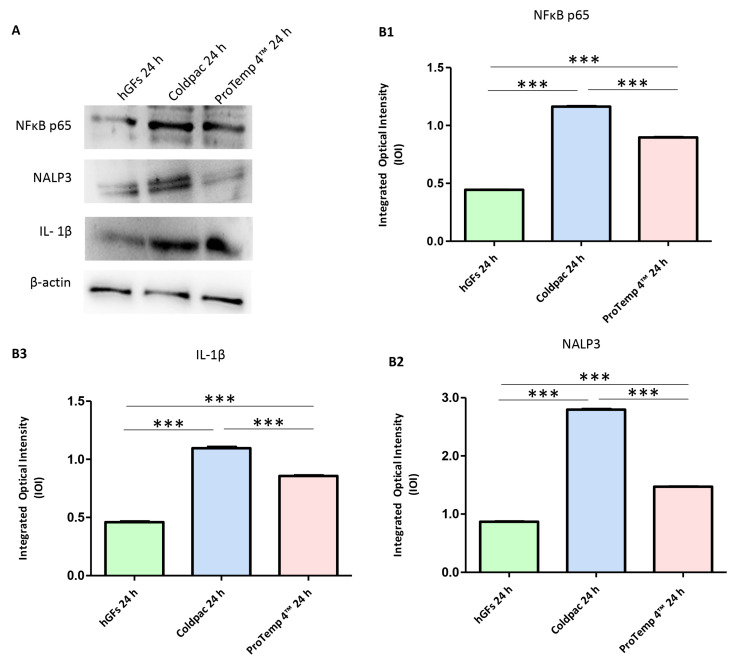
Western blotting analysis. NFκB p65, NLRP3 and IL-1β protein expression were evaluated in hGFs alone, hGFs cultured with Coldpac (Yates Motloid) and hGFs cultured with ProTemp 4™ (3M ESPE ™) for 24 h (**A**) Densitometric measurements of protein bands expressed (**B1**–**B3**). Error bars: standard deviation (±SD). *** *p* < 0.001. Loading control: β-actin.

**Figure 8:**
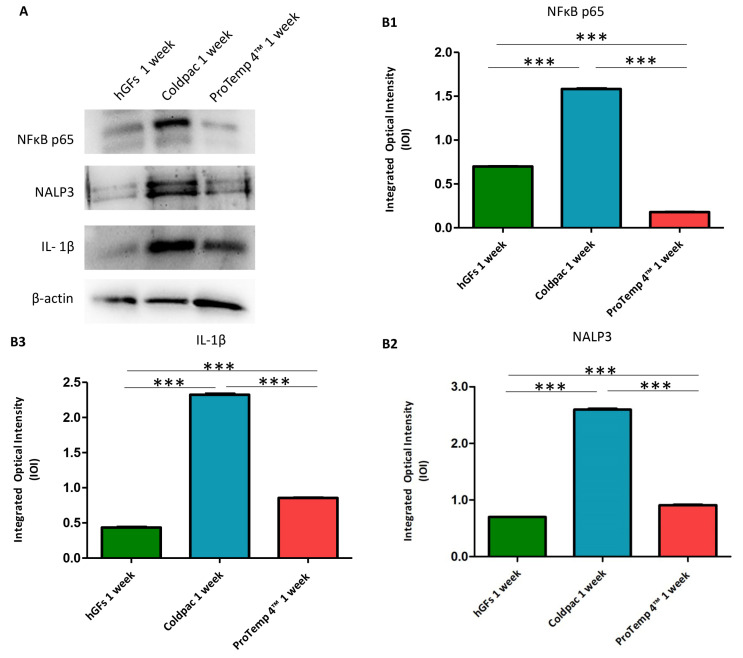
Western blotting analysis. NFκB p65, NLRP3 and IL-1β protein expression were evaluated in hGFs alone, hGFs cultured with Coldpac (Yates Motloid) and hGFs cultured with ProTemp 4™ (3M ESPE ™) for 1 week. (**A**) Densitometric measurements of protein bands expressed (**B1**–**B3**). Error bars: standard deviation (±SD). *** *p* < 0.001. Loading control: β-actin.

**Figure 9:**
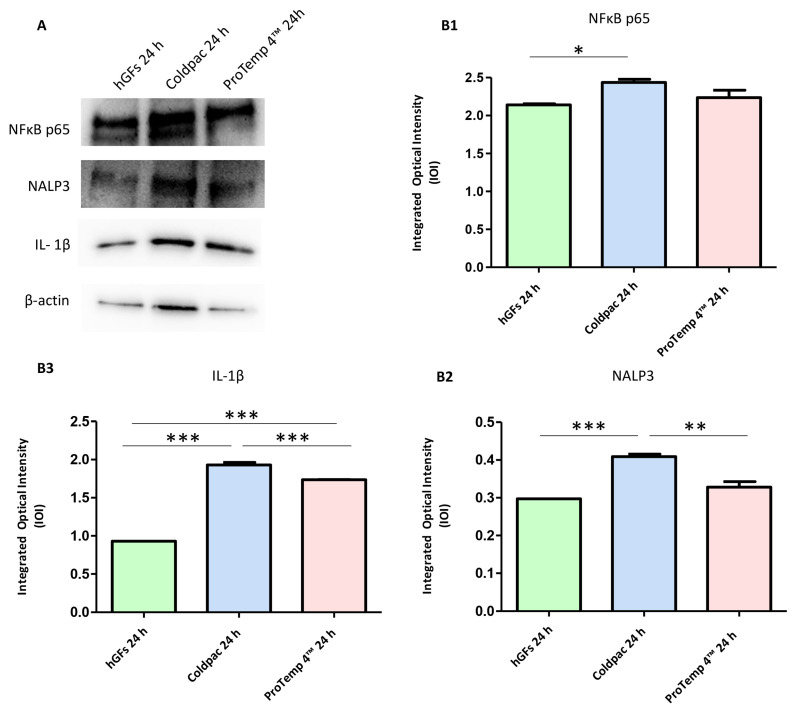
Western blotting analysis. NFκB p65, NLRP3 and IL-1β protein expression were evaluated in hGFs alone, hGFs cultured with eluate derived from Coldpac (Yates Motloid) and hGFs cultured with eluate derived from ProTemp 4™ (3M ESPE ™) for 24 h (**A**) Densitometric measurements of protein bands expressed (**B1**–**B3**). Error bars: standard deviation (±SD). * *p* < 0.05; ** *p* < 0.01; *** *p* < 0.001. Loading control: β-actin.

**Figure 10:**
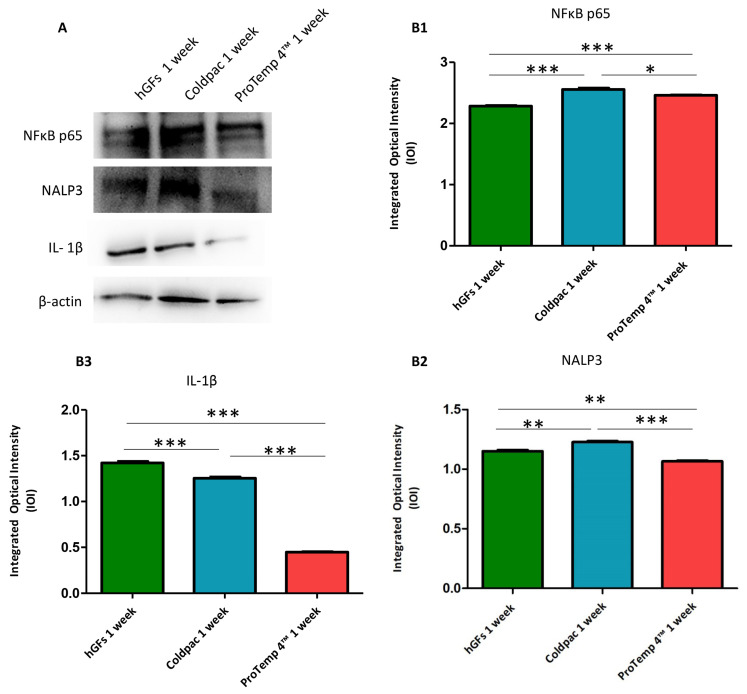
Western blotting analysis NFκB p65, NLRP3 and IL-1β protein expression in hGFs alone, hGFs cultured with eluate derived from Coldpac (Yates Motloid) and hGFs cultured with eluate derived from ProTemp 4™ (3M ESPE ™) for 1 week. (**A**) Densitometric measurements of protein bands expressed (**B1**–**B3**). Error bars: standard deviation (±SD). * *p* < 0.05; ** *p* < 0.01; *** *p* < 0.001. Loading control: β-actin.

The immunofluorescence figures showed the expression of NFκB p65/NLRP3/IL-1β in hGFs alone, cultured with Coldpac (Yates Motloid) and cultured with ProTemp 4™ (3M ESPE ™) and their eluate for 24 h and 1 week. The data evidenced that the NFκBp65/NLRP3/IL-1β pathway was significantly expressed in hGFs cultured with Coldpac (Yates Motloid) and its eluate after 24 h and 1 week of culture compared to hGFs cultured with ProTemp 4™ (3M ESPE ™) and its eluate, and hGFs alone (Figure 3, Figure 4, Figure 5 and Figure 6). Western blot analysis data confirmed the results obtained by immunofluorescence analysis (Figure 7, Figure 8, Figure 9 and Figure 10).

### 2.4. Genes Expression

**Figure 11:**
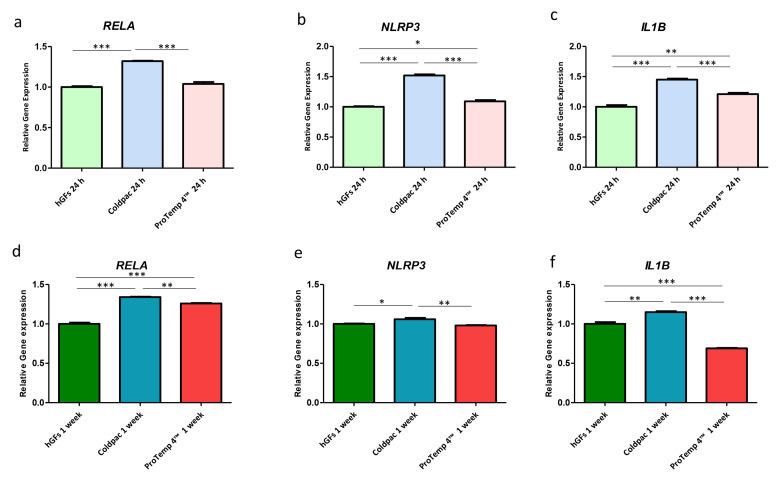
(**a**–**f**) Histograms of RT-PCR show the mRNA levels of NFκB p65/NLRP3/IL-1β in hGFs cultured alone, hGFs cultured with Coldpac (Yates Motloid), and hGFs cultured with ProTemp 4™ (3M ESPE ™) for 24 h and 1 week. * *p* < 0.05; ** *p* < 0.01; *** *p* < 0.001.

**Figure 12:**
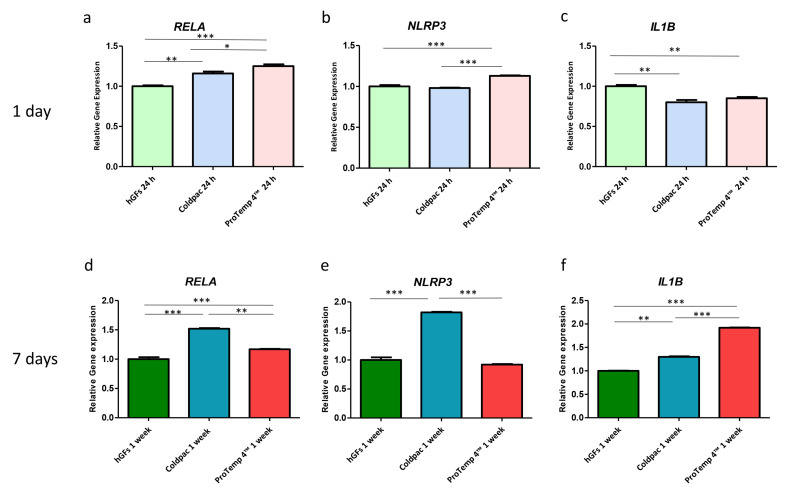
(**a**–**f**) Histograms of RT-PCR show the mRNA levels of NFκB p65/NLRP3/IL-1β in hGFs cultured alone, hGFs cultured with eluate derived from Coldpac (Yates Motloid), and hGFs cultured with eluate derived from ProTemp 4™ (3M ESPE ™) for 24 h and 1 week. * *p* < 0.05; ** *p* < 0.01; *** *p* < 0.001.

The histograms show the gene expression of NFκB p65/NLRP3/IL-1β evaluated by real-time PCR in hGFs cultured alone, with Coldpac (Yates Motloid), and with ProTemp 4™ (3M ESPE ™) and their eluate for 24 h and 1 week (Figure 11 and Figure 12). These results obtained by real-time PCR confirm the data obtained from CLSM and Western Blot analyses.

## 3. Discussion

Coldpac (Yates Motloid) is a PMMA self-curing resin composed by polymer powder and monomer liquid. The incomplete polymerization reaction can lead to a monomer release in the oral cavity. As reported in other studies, monomer release can cause deleterious effect on the oral mucosa and cell metabolism [29,30,31]. To date, no studies have reported the biological effects of Coldpac (Yates Motloid) resin in the oral cavity. This resin has been studied to understand its marginal precision in direct crowns reconstruction and its fracture resistance [32,33].

The more recent ProTemp 4 ™ (3M ESPE ™) is an autopolymerizing provisional resin based on Bis-GMA. Several reports have emphasized its strength, mechanical properties and biocompatibility [34,35,36,37,38]. Like Coldpac (Yates Motloid) resin, ProTemp 4™ (3M ESPE ™) can release some monomers due to the incomplete polymerization process; however, the toxicity is lower than that of other resins [19]. In prosthodontics, the provisional restorations are used to protect and maintain the function and appearance of teeth until a permanent restoration can be placed [39]. In general, the elapsed time between the provisional restoration insertion and the definitive restoration placement has a mean of one week, depending on the complexity and number of elements to be restored. The present work aimed to understand the better adhesion and biocompatibility properties of Coldpac (Yates Motloid) resin, ProTemp 4™ (3M ESPE ™) resin, and their eluates at different time points in an in vitro model of hGFs. In the present study, the morphology and the adhesion of hGFs were evaluated by scanning electron microscopy and confocal laser scanning microscopy. As mentioned earlier, the resin monomers release can impact negatively the health and the integrity of the biological width, which may lead into gingival recession and inflammation [40]. The biological width is composed of the epithelium and connective tissues and acts as a dividing line between the periodontal tissue and the oral cavity [41]. In order to maintain a proper biological width provisional, resin must have biocompatibility and the ability to allow the adhesion of gingival cells. Accordingly, we have studied the inflammatory pathway in hGFs with respect to the exposure of the resins. The inflammatory process was studied by analyzing the modulation of the NFκB p65/NLRP3/IL-1β pathway to understand the effects of monomers released by the resins’ surfaces. Activation of the NLRP3 inflammasome is mediated primarily by NFkB which, in response to various PRR ligands and cytokines, acts as a transcription factor inducing the expression of NLRP3 and pro-IL-1β [42]. Both the pro-IL-1β gene and the NLRP3 gene contain NF-κB binding sites in the promoter region [43,44].

Inflammation leads to the activation of the inflammasome through the overexpression of its components: NLRP3, caspase 1 and pro-IL-1β, which can be induced by pathogen-associated molecular patterns (PAMP), or damage-associated molecular patterns (DAMP), through pattern-recognition receptors (PRRs) such as TLRs. Our in vitro study suggested that after 24 h and 1 week, hGF cells cultured in adhesion and with eluate derived from ProTemp 4™ (3M ESPE ™) resin evidenced a downregulation in the expression of the inflammatory mediators, such as NFκB p65, NLRP3, and IL-1β, compared to the cells cultured with Coldpac (Yates Motloid).

In addition, the cells cultured with ProTemp 4™ (3M ESPE ™), after 24 h and 1 week of culture, reported a higher cell viability compared to the cells cultured with Coldpac (Yates Motloid), as established with MTS cell analysis. Similar results were obtained when hGFs were placed in culture with the eluate derived from ProTemp 4™ (3M ESPE ™) resin showing a higher cell viability compared to the cells cultured with eluate derived from Coldpac (Yates Motloid). Moreover, the evaluation of protein expression of Ki-67, a principal marker involved in cell proliferation, was evaluated by confocal microscopy. The data showed a significantly higher expression in cells cultured with ProTemp 4™ (3M ESPE ™) compared to the cells cultured with Coldpac (Yates Motloid). As regards the evaluation of eluate effects, eluate derived from ProTemp 4™ (3M ESPE ™) resin showed a higher expression of Ki-67 compared to the hGFs cultured with eluate derived from Coldpac resin (Yates Motloid). The gene expression of NFκB p65, NLRP3, and IL-1β were also evaluated and confirmed the results obtained in confocal microscopy. In conclusion, it can be inferred that ProTemp 4™ (3M ESPE ™) exhibited favorable biocompatibility properties, indicating its potential for use in clinical applications when compared to Coldpac (Yates Motloid), suggesting a better performance in terms of cells–material interaction.

## 4. Material and Methods

### 4.1. Resins

Resin specimens were obtained by using two glass plates spaced 3 mm apart in order to obtain a smooth surface and a controlled thickness. ProTemp™ 4 (Protemp™ 4 Temporisation Material, 3M ESPE, St. Paul, MN, USA) (composition: 2,2′-[(1-methylethylide)bis(4,1-phenyleneoxy)] bisethyl diacetate, 1-benzyl-5-phenylbarbituric acid, silanamine, 1,1,1-trimethyl-N-(trimethylsilyl)-, 3,5,5-trimethylperoxyhexanoate of tert-butyl, bisphenol A-polyethylene glycol dieteres dimethacrylate, amorphous silica (7631-86-9), surface-modified with 2-propenoic acid, methyl-, 3-(thyrmethoxysilyl) propyl ester (2530-80-0) and phenyltrimethoxysilane (2996-92-1), reaction products of hexamethylene-1,6-diisocyanate, oligomers with 6-hydroxyhexanoate of 2-[(2-methyl-1-oxoally)oxy]ethyl and 2-hydroxyethyl methacrylate, silanamine, 1,1,1-trimethyl-N-(trimethylsilyl)-, silica hydrolysis products) and Coldpac (Coldpac tooth acrylic, Yates Motloid, Chicago, IL, USA) (composition: methyl methacrylate, 2-propenoic acid, 2-methyl-, 1,2-ethanediyl ester, Colorstable agent, ultraviolet light absorber aromatic ketone, Cross lining agent polyfunctional acrylic monomer, benzanamine, N,N,4-trimethyl-,) were mixed and polymerized following the manufacturer’s instructions. Using a 5mm diameter Trephine bur, 50 specimens were obtained for each type of resin. After the trimming process, disks were put in an ultrasonic bath with distilled water for 30 min to remove any trimming remnants. All disks were sterilized using an autoclave (Anthos A22, Cefla s.c.Via Selice Provinciale, 23/a 40026 Imola-Bo (Italy) at 134 °C for 50 min. After the drying program, specimens were immediately placed in cell culture.

### 4.2. Sample Preparations for Eluates Deriving from Protemp 4™ (3M ESPE ™) and Coldpac Resins (Yates Motloid)

ProTemp 4™ (3M ESPE ™) and Coldpac (Yates Motloid) resins were mixed and polymerized following the manufacturer’s instructions in order to obtain round section bars of 10 mm in diameter. From the bars, 30 disks were then obtained for each material with a thickness of 2 mm, using a water-cooled diamond disk microtome (TT System; TMA2, Grottammare, Italy). The disks’ surfaces were finished with 1200 grit paper and subsequently rinsed in Marseille soap and water in an ultrasonic bath (500 W for 5 min at 37 °C) to remove the finishing impurities; 100% isopropyl alcohol was used for 1 min to disinfect and then the disks were rinsed and air dried followed by another disinfection in ethanol for 45 min and rinsing in 8 mL of sterile water three times under a sterile hood with the aim of eliminating any pathogenic component from the surface of the samples.

15 disks from each group were placed in 50 mL falcons (two copies for each material) in 19 mL of DMEM; the falcons were sealed and placed in an oven at 37 °C at 100% humidity for 1 week. The eluate was then removed from the samples and placed in 50 mL falcons and left at 4 °C awaiting future use. The eluate was then mixed with the culture medium in a ratio of 1:1 and subsequently added to the plates containing the hGFs.

### 4.3. Cell Culture

hGFs (PCS-201-018 ATCC, Manassas, VA, USA) were cultured in Dulbecco’s Modified Eagle’s Medium (DMEM, Lonza Walkersville, MD, USA) with the addition of 10% Fetal Bovine Serum and 0.1% gentamicin [45] and maintained in an incubator at 37 °C in a humidified atmosphere with 5% CO_2_ and 95% air. At 75–80% confluence, the cells were expanded producing subcultures.

### 4.4. Experimental Study Design

hGFs were cultured alone and/or with resins Coldpac (Yates Motloid) and Protemp 4™ (3M ESPE ™) and/or with eluates derived from Coldpac (Yates Motloid) and Protemp 4™ (3M ESPE ™) resins. The experimental points were performed in triplicate. 

The hGFs cultured alone and/or with resins Coldpac (Yates Motloid) and Protemp 4™ (3M ESPE ™)

- hGFs cultured alone for 24 h
- hGFs cultured alone for 1 week
- hGFs cultured with Coldpac (Yates Motloid) for 24 h
- hGFs cultured with Coldpac (Yates Motloid) for 1 week
- hGFs cultured with ProTemp 4™ (3M ESPE ™) for 24 h
- hGFs cultured with ProTemp 4™ (3M ESPE ™) for 1 week

The hGFs cultured alone and/or with eluates derived from Coldpac (Yates Motloid) and Protemp 4™ (3M ESPE ™) resins

- hGFs cultured alone for 24 h
- hGFs cultured alone for 1 week
- hGFs cultured with the eluate derived from ProTemp 4™ (3M ESPE ™) for 24 h
- hGFs cultured with the eluate derived from ProTemp 4™ (3M ESPE ™) for 1 week
- hGFs cultured with the eluate derived from Coldpac (Yates Motloid) for 24 h
- hGFs cultured with the eluate derived from Coldpac (Yates Motloid) for for 1 week

### 4.5. Cell Metabolic Activity

The 3-(4,5-dimethylthiazol-2-yl)-5-(3-carboxymethoxyphenyl)-2-(4-sulfo-phenyl)-2H-tetrazolium (MTS) assay (CellTiter 96^®^ Aqueous One Solution Cell Proliferation Assay, Promega, Madison, WI, USA) was used to evaluate the cell metabolic activity of hGFs cultured alone and/or with resins Coldpac (Yates Motloid) and Protemp 4™ (3M ESPE ™) and/or with eluates derived from Coldpac (Yates Motloid) and Protemp 4™ (3M ESPE ™) resins. For the MTS assay, 2.4 × 10^3^ hGFs/well were seeded into 96-well plates and maintained in culture at 37 °C with Fibroblast Basal Medium (ATCC PCS-201-030) supplemented with Fibroblast Growth Kit-Low Serum (ATCC PCS-201-041) for 24 h and 1 week. After these respective culture times, 20 μL/well of MTS staining solution was added and the plates were incubated at 37 °C for 3 h. Quantification of formazan salts, directly related to cell viability, was measured through absorbance at a wavelength of 490 nm using the Synergy™ HT Multi-detection microplate reader (Biotech, Winooski, VT, USA) [46]. The MTS assay was performed in triplicate.

### 4.6. SEM Analysis

SEM analysis allowed the morphological evaluation of hGFs cultured alone, hGFs cultured with ProTemp 4™ (3M ESPE ™) resin and hGFs cultured with Coldpac (Yates Motloid) resin for 24 h and for 7 days. The same morphological evaluation was performed for hGFs cultured individually, hGFs cultured in contact with ProTemp 4™ (3M ESPE ™) resin, and hGFs cultured in contact with Coldpac (Yates Motloid) resin for 24 h and for 7 days. The preparation of the samples was made according to the following protocol: The cells, after 24 h and 1 week of culture, were fixed for 1 h at room temperature in 2.5% glutaraldehyde, rinsed three times with distilled H_2_O and incubated for 1 h with 1% osmium tetroxide at room temperature. The dehydration was performed with different concentrations of ethanol (60%, 80%, 95%, and 100%) and subsequently air dried [47]. They were mounted on aluminum stubs and sputtered with gold in Emitech K550 (Emitech Ltd., Ashford, UK) [48]. Morphological analysis was performed using a high-resolution SEM (ZEISS EVO 50, Jena, Germany).

### 4.7. Confocal Laser Scanning Microscope (CLSM)

hGFs were cultured in 8-well culture slides (Corning, Glendale, AZ, USA) (density of 6.4 × 10^4^/well). 24 h after the treatment, fixation with paraformaldehyde (PFA) (BioOptica, Milan, Italy) diluted at 4% in 0.1 M in PBS (Lonza, Basel, Switzerland) followed for one hour at room temperature. Once fixation was completed, the cells were washed in PBS three times and permeabilized with 0.1% Triton X-100 (BioOptica) in PBS for 6 min. They were subsequently washed in PBS and saturated with 5% skimmed milk in PBS for 1 h at room temperature. Saturation was followed by overnight incubation at 4 °C with primary antibodies diluted 2.5% in PBS as follows: 1:200 anti-NFκB p65 (sc-8008, Santa Cruz Biotechnology, Dallas, TX, USA), 1:200 anti-NLRP3 (sc-134306, Santa Cruz Biotechnology), 1:200 anti-IL-1β (sc-32294, Santa Cruz Biotechnology), 1:200 anti-Ki-67 (MA5-15690, Invitrogen, Eugene, OR, USA) and 1:200 Anti-TOMM20 (sc-17764, Santa Cruz Biotechnology). The following morning, 1 h incubation at 37 °C with Alexa Fluor 568 red fluorescence-conjugated goat anti-mouse secondary antibody (A11031, Invitrogen) 1:200 in 2.5% skimmed milk on PBS. Nuclei and cytoskeletal actin and nuclei were highlighted with TOPRO (T3605, Invitrogen) and Alexa Fluor 488 phalloidin green fluorescent conjugate (A12379, Invitrogen) respectively with a 1:200 incubation in 2.5% skimmed milk % in PBS for 1 h at 37 °C. Immunofluorescence was evaluated with image acquisition using the Zeiss LSM800 confocal system (Carl Zeiss, Jena, Germany) [49].

### 4.8. Western Blotting Analysis 

The proteins derived from hGF lysis were loaded (50 µg) onto a polyacrylamide gel and subjected to an electrophoretic run. With the SEMI-dry blotting apparatus (Bio-Rad Laboratories Srl, Milan, Italy) the proteins were transferred onto a polyvinylidene fluoride (PVDF) membrane which, once the transfer was complete, was subjected to saturation with 5% skimmed milk in PBS 0.1% Tween-20 (Sigma-Aldrich, Saint Louis, MO, USA) and then incubated overnight at 4 °C with the following primary antibodies: anti-NFκB p65 (1:500) (sc-8008, Santa Cruz Biotechnology), anti-NLRP3 (1:500) (sc-134306, Santa Cruz Biotechnology), anti-IL-1β (1:500) (sc-32294, Santa Cruz Biotechnology) anti-Ki-67 (1:500) (MA5-15690, Invitrogen) and Anti-TOMM20 (1:500) (sc-17764, Santa Cruz Biotechnology). β-Actin (1:750) (sc-47778, Santa Cruz Biotechnology) was used as a loading control. Subsequently for the detection of primary antibodies, the membranes were incubated for 1 h at room temperature with peroxidase-conjugated goat anti-mouse secondary antibody (A90-116P, Bethyl Laboratories Inc., Montgomery, TX, USA) 1:5000 diluted in 2.5% skimmed milk in PBS and 0.1% Tween-20 following three washes with PBS 0.1% Tween-20. Chemiluminescence determined by protein expression levels was detected with Alliance 2.7 (Uvitec, Cambridge, UK). The densitometry values obtained were normalized with those of β-actin. The analysis was carried out in triplicate [50].

### 4.9. RNA Isolation and Real-Time RT-PCR Analysis

Total RNA, extracted using PureLink RNA Mini Kit (Ambion, Thermo Fisher Scientific, Milan, Italy) was used to evaluate NFκB p65, NLRP3, and IL-1β mRNA expression by real-time PCR. Then, one microgram of total RNA was reverse transcribed using M-MLV reverse transcriptase (M1302 Sigma-Aldrich) to synthesize cDNA for 10 min at 70 °C, 50 min at 37 °C, and 10 min at 90 °C. Three independent biological replicates were analyzed for each sample. Real-time PCR was performed with the Mastercycler real plex real-time PCR system (Eppendorf, Hamburg, Germany). Beta-2 microglobulin (B2M Hs.PT.58v.18759587, Tema Ricerca Srl) was used as an endogenous marker for data normalization. The expression levels of all mRNAs considered were evaluated in hGF cells cultured alone, in hGF cells cultured with Coldpac (Yates Motloid) and hGF cells cultured with ProTemp 4™ (3M ESPE ™) for 24 h and 1 week. Commercially available PrimeTime™ RELA (Hs.PT.58.22880470, Tema Ricerca Srl), NLRP3 (Hs.PT.58.39303321, Tema Ricerca Srl), IL-1β (Hs.PT.58.1518186) and PrimeTime™ Gene Expression Master Mix (cat. n°1055772, Tema Ricerca Srl) were used according to standard protocols (Table 1). Expression levels for each gene were obtained according to the 2^−ΔΔCt^ method ((relative quantification) [51]. Real-time PCR was performed in three independent experiments.

### 4.10. Statistical Analysis

GraphPad 5 software (GraphPad, San Diego, CA, USA) with one-way ANOVA allowed evaluation of the statistical significance of the data using a post hoc Tukey’s multiple comparisons analysis. Values of *p* < 0.05 were considered statistically significant.

## 5. Conclusions

The results obtained in this study demonstrate that there are differences between Coldpac (Yates Motloid) and ProTemp 4™ (3M ESPE ™) resins in terms of cell viability, proliferation, and biological responses. Cell viability was significantly reduced in hGFs cultured with Coldpac compared to hGFs cultured alone or with ProTemp 4™, both at 24 h and one week, as well as cell proliferation. Furthermore, hGFs cultured in Coldpac resin showed an increased inflammatory response via the NFκBp65/NLRP3/IL-1β pathway compared to hGFs cultured in ProTemp4™.

## Figures and Tables

**Table 1:** Primer sequences used for real-time PCR reactions.

Gene	Forward Primer Sequence (5′-3′)	Reverse Primer Sequence (5′-3′)
*RELA*	5′-CGAGCTTGTAGGAAAGGACTG-3′	5′-TGACTGATAGC-CTGCTCCAG-3′
*NLRP3*	5′-GAATGCCTTGG-GAGACTCAG-3′	5′-AGATTCTGATT-AGTGCTGAGTACC-3′
*IL-1β*	5′-CGTCCTAAAGA-CTCCATGATCTG-3′	5′-ACCAATCTTGT-AGGACTGACC-3′
*B2M*	5′-GGACTGGTCTT-TCTATCTCTTGT-3′	5′-ACCTCCATGAT-GCTGCTTAC-3′

## Data Availability

Data are available to the corresponding author upon request.
